# Metabolite transport and associated sugar signalling systems underpinning source/sink interactions

**DOI:** 10.1016/j.bbabio.2016.07.007

**Published:** 2016-10

**Authors:** Cara A. Griffiths, Matthew J. Paul, Christine H. Foyer

**Affiliations:** a Plant Biology and Crop Science, Rothamsted Research, Harpenden, Hertfordshire AL5 2JQ, UK; b Centre for Plant Sciences, School of Biology, Faculty of Biological Sciences, University of Leeds, Leeds LS2 9JT, UK

**Keywords:** Phloem loading, Redox regulation, Source-sink interactions, Sucrose transporters, Sugar signalling, Trehalose

## Abstract

Metabolite transport between organelles, cells and source and sink tissues not only enables pathway co-ordination but it also facilitates whole plant communication, particularly in the transmission of information concerning resource availability. Carbon assimilation is co-ordinated with nitrogen assimilation to ensure that the building blocks of biomass production, amino acids and carbon skeletons, are available at the required amounts and stoichiometry, with associated transport processes making certain that these essential resources are transported from their sites of synthesis to those of utilisation. Of the many possible posttranslational mechanisms that might participate in efficient co-ordination of metabolism and transport only reversible thiol-disulphide exchange mechanisms have been described in detail. Sucrose and trehalose metabolism are intertwined in the signalling hub that ensures appropriate resource allocation to drive growth and development under optimal and stress conditions, with trehalose-6-phosphate acting as an important signal for sucrose availability. The formidable suite of plant metabolite transporters provides enormous flexibility and adaptability in inter-pathway coordination and source-sink interactions. Focussing on the carbon metabolism network, we highlight the functions of different transporter families, and the important of thioredoxins in the metabolic dialogue between source and sink tissues. In addition, we address how these systems can be tailored for crop improvement.

## Highlights

• Metabolite transport facilitates inter-pathway regulation, cell to cell communication and co-ordination of source and sink processes.
• Highlighting the carbon metabolism network, this review focuses on the role of transporters, carbon flow, and signalling.- The integration of sucrose and trehalose pathways as nodes in the regulation of plant growth and signalling is approached.

## Graphical abstract

Metabolite transport not only allows the flawless coordination of pathways in different organelles and between cells, but it is also balances energy provision and utilisation throughout the whole plant. Plant metabolite transport displays enormous plasticity and flexible regulation. Focussing particularly on carbon transporters, this review discusses transport functions in source and sinks organs, with sucrose and trehalose in source-sink communication that encompasses sophisticated perception and signalling systems.

**Figure:**
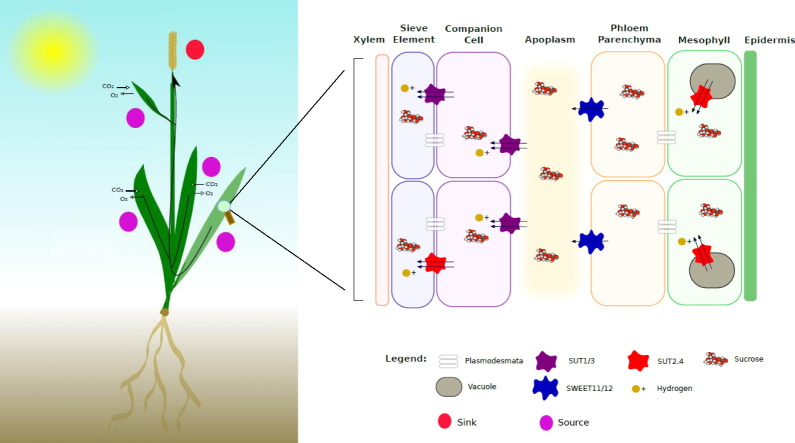

## 1 Introduction

Plant metabolism is driven by the energy-transducing reactions of the chloroplasts and mitochondria, which use ATP, reducing power {NAD(P)H)} and associated metabolites as the major currency of energy exchange. Plant cells synthesize all of the metabolic building blocks for growth, biomass production and defence such as sugars and carbohydrate polymers, lipids, amino acids and secondary metabolites such as alkaloids and terpenoids. Metabolite fluxes through parallel pathways often occur simultaneously in differing cellular compartments [1]. Moreover, the metabolic requirements of different developmental and defence processes change dynamically with time and according to prevailing environmental conditions, requiring overlapping layers of short and long-term regulation. Dynamic regulation of photosynthetic and respiratory metabolism involving extensive metabolite exchange provides tight but flexible delivery of the correct building blocks in appropriate amounts at the right time and place. While our understanding of the co-ordination of the pathways of primary carbon and nitrogen assimilation has greatly increased in recent decades, relatively little is known about regulation of the essential transport systems between compartments, cells and different plant organs [2], [3], [4]. Metabolites such as sugars and amino acids occupy central positions in the coordination of processes in different cellular compartments, facilitating multiple points of reciprocal control between pathways [5], [6], [7]. In addition, metabolites, such as sucrose and nitrate act as signals regulating gene expression to optimise pathway fluxes according to prevailing environmental conditions. In this review we discuss the importance of transporters in the metabolic coordination network within cells, between cells and between organs, with a particular focus on carbon metabolism and sugar transport and signalling, and the main pathways that interact with carbon metabolism to ensure appropriate provision of resources between source and sink organs.

## 2 Carbon/nitrogen interactions

The efficient operation of carbon metabolism leading to carbon gain intrinsically depends on the successful uptake of other nutrients particularly nitrogen and phosphorus. Increasing focus is being placed on this interdependence because of the uncertainties that persist regarding how plant yields will be influenced by climate change and the increasing availability of carbon dioxide in the atmosphere. On one hand, studies in FACE (Free-Air CO_2_ Enrichment) systems have demonstrated that nitrogen use efficiency will increase as atmospheric carbon dioxide becomes more available [8]. Conversely, the concomitant inhibition of photorespiration will have an adverse effect on primary nitrogen assimilation because of limitations on redox cycling [9]. In addition, for future agriculture to be sustainable it is crucial that further yield gains are achieved with current and preferably low levels of soil fertilization [10]. Nitrogen use efficiency (NUE) is a highly complex trait involving N uptake efficiency (NUpE) and N assimilation efficiency (NUtE). NUpE is influenced by root architecture and the activities of large families of NO_3_^−^ and NH_4_^+^ transporters [10]. Several families of nitrate transporters (NRT1, NRT2 and CLC) mediate the uptake and transport of nitrate in plants [10]. In general, NRT2 transporters have a high affinity for nitrate, while most of the NRT1 family have a low affinity for nitrate. Perhaps the best characterised example is NRT1.1, which is a dual affinity transporter. It is switched from low- to high-affinity transport forms by phosphorylation of Thr101 [10]. The post-translational modification of NRT1.1 enhances the affinity of the protein for nitrate, while high nitrate also acts as a transcriptional suppressor of NRT1.1expression. NRT1.1 is an important nitrate-sensing component that regulates lateral root development [11] by facilitating auxin transport [12]. This transporter also participates in the control of the expression of genes such as the high-affinity nitrate transporter, NRT2.1, whose expression is also regulated by nitrate availability [13], [14], [15].

Nitrate reduction in the cytosol is catalysed by NADH-dependent nitrate reductases (NR). While the activity of this enzyme is regulated in response to environmental and metabolic triggers as well as protein phosphorylation, the rate of nitrate assimilation can be limited by the availability of NADH [2], [3], [16], [17]. Metabolite transport between the chloroplasts and cytosol is important in boosting the cytosolic NADH pool, involving dicarboxylates transport and shuttle systems for malate and oxaloacetate. The 2-oxoglutarate/malate transporter, AtpOMT1 plays an important role in this process, functioning both as an oxaloacetate/malate transporter in the malate valve pathway and as a 2-oxoglutarate/malate transporter mediating the transfer of carbon skeletons [18], [19].

Nitrite generated by the action of NR is transported into the chloroplasts where it is reduced by nitrite reductase (NiR) to ammonium (NH_4_^+^), which is assimilated into amino acids by the glutamine synthetase/glutamine-2-oxoglutarate aminotransferase (GS-GOGAT) pathway. Thereafter, a raft of aminotransferases and other enzymes catalyse transfer of the amino group to form other amino acids. Provision of the 2-oxoglutarate required for ammonia assimilation requires partial operation of the TCA cycle in the mitochondria [3].

## 3 Pathway co-ordination of by reversible thiol-disulphide exchange

**Fig. 1:**
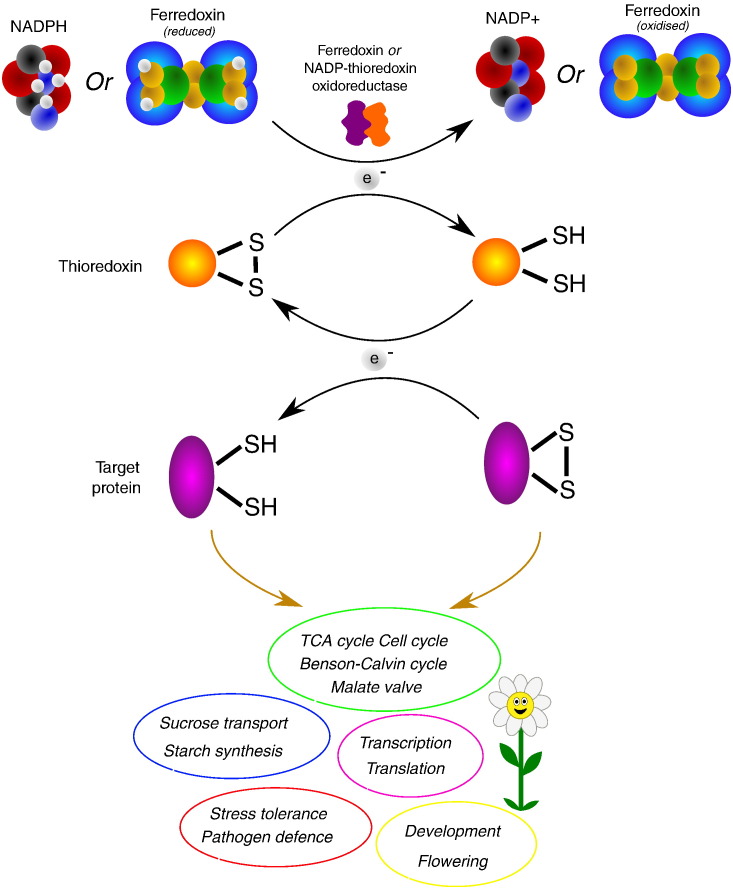
The central role of thiol-disulphide exchange regulation via thioredoxins in plant biology.

The extensive cycling of reducing equivalents facilitated by metabolite transport [9], [20], [21] also facilitates pathway co-ordinates through influences on post-transcriptional protein modifications (PTM) that provide dynamic and reversible protein processing to modulate enzyme activity, binding properties and function. Many types of PTM (over 450) have been identified to date. Many proteins involved in carbon and nitrogen metabolism are subject to PTMs such as protein acetylation, succinylation, malonylation, butyrylation, and propionylation. For example, the large subunit of ribulose-1, 5-bisphosphate carboxylase (RuBisCO) is extensively succinylated and acetylated. Deacetylation of the RuBisCO protein has been shown to activate the enzyme [22]. However, in most cases the functional significance of these PTMs has yet to be resolved. In contrast, the functions of protein phosphorylation and thiol-disulphide exchange processing (Fig. 1) have been extensively characterised. For example, the light and thiol-dependent activation of photosynthetic CO_2_ fixation pathway enzymes requires thioredoxins (TRX), which are small proteins with disulphide reductase activities [23]. TRX reductases in the stroma reduce TRXs using either reduced ferredoxin or NADPH produced by the photosynthetic electron transport chain [24]. Conversely, TCA cycle enzymes such as succinate dehydrogenase and fumarase are reductively inactivated by TRX [25], [26], [27]. In this way, the TRX systems in the plastids, mitochondria and cytosol link photosynthetic and respiratory electron transport activities to functional changes in enzyme activities [23], [28].

The photosynthetic electron transport chain provides the reductive “push” that keeps the stromal TRXs reduced in the light, ensuring that the activation states of the thiol modulated enzymes involved in CO_2_ fixation are matched to rate of production of reduced ferredoxin and NADPH [24], [29], [30], [31]. When this push is removed in the dark, the TRXs revert to their oxidized forms, which in turn allow oxidative inactivation of the thiol-modulated enzymes. In addition, chloroplast TRXs also regulate malate and oxaloacetate transport [32] and starch metabolism through effects on adenosine diphosphate (ADP)-glucose pyrophosphorylase (AGPase). The small subunit of this key enzyme of starch biosynthesis is regulated by redox-dependent dimerization in response to sugar availability [33], [34], [35].

Appropriate resource allocation between different plant organs might also be achieved by post-translational modification of sucrose transporters. Sucrose export from leaves occurs both in the light and dark to ensure the continuous and stable provision of adequate carbon resources to drive plant growth and development. The transport of sucrose is mediated by membrane-localised sucrose transporters, whose properties will be discussed later in detail. The activities of these transporters are regulated by changes in cellular redox status and by protein-protein interactions [36]. For example, SUT1 interacts with a small cysteine-rich cell wall protein in potato called SN1 [37]. The SN1 protein belongs to the Snakin/gibberellic acid stimulated (GAS) family in *Arabidopsis* [38], [39]. Silencing of SN1 leads to perturbations in cellular redox metabolism, particularly antioxidant activities [39]. SUT1 is able to form dimers with a protein disulphide isomerase in a redox-dependent interaction and is hence able to interact with other proteins that are involved in metabolism or secretion [40]. However, no increases in the transport activity of StSUT1 have been shown to be dependent on this dimerization [41].

## 4 Transport of sugars between the chloroplasts and cytosol

Triose phosphate/phosphate, glucose, maltose transporters are important mediators of carbon transfer between the plastids and cytosol [7]. Carbon assimilated during photosynthesis is either transported out of the chloroplast or used for starch biosynthesis in the stroma. Carbon is exported from the chloroplasts in the light as triose phosphate or 3-phosphoglyceric acid. This occurs across the triose-phosphate/phosphate translocator (TPT) in strict stoichiometric exchange for inorganic phosphate (Pi). TPT belongs to the plastidial phosphate translocator family, which has three other members: the pentose phosphate/phosphate translocator (XPT), phosphoenolpyruvate (PEP)/phosphate translocator (PPT) and the glucose 6-phosphate/phosphate translocator (GPT).

TPT is relatively abundant in the chloroplast inner membrane, constituting 10–12% of the protein content [42]. Triose-phosphates transported by TPT are utilised for sucrose synthesis in the cytosol or for the generation of organic acids through the anaplerotic pathway. Inorganic phosphate produced during sucrose synthesis is transported back into the chloroplast via the TPT to be used in ATP synthesis [43], [44], [45]. Therefore, the re-cycling of phosphate maintains photosynthetic electron transport and the pentose phosphate pathways [46]. While transgenic potato plants that have reduced TPT levels or where TPT is knocked out completely show no phenotype, the chloroplasts have a reduced capacity to import phosphate by up to 30%, together with a 40–60% reduction in maximal photosynthesis [47]. The TPT is therefore central to the regulation of carbon partitioning between starch and sucrose [48], [49]. Transgenic tobacco plants that over-express TPT did not show a marked phenotype or any marked effects on amino acid production [50]. However, they incorporated more CO_2_ into sucrose and had higher leaf starch/sucrose ratios than the wildtype [50]. Transgenic *Arabidopsis* lines over-expressing TPT and also fructose-1, 6-bisphosphatase had higher photosynthetic carbon assimilation rates. In addition, these lines also accumulated more sucrose, glucose and fructose than controls and showed enhanced growth compared to the wild type [51].

Another type of phosphate transporter (PPT) facilitates the transport of three-carbon compounds containing a phosphate group at the C2 position, such as phospho*enol*pyruvate (PEP) and 2-phosphoglyceratefor phosphate across the inner plastid envelope membrane in heterotrophic tissues [52]. PPT transporters are essential for the transport of PEP into plastids, which with the exception of lipid-storage tissues, cannot produce PEP [53]. Of the two genes encoding PPTs in *Arabidopsis*, *AtPPT2* is expressed in leaves and *AtPPT1* transcripts are localised only in the vasculature. Analysis of *Atppt1* mutants demonstrated that this transporter is essential for plant development and metabolism [54]. In addition, *AtPPT1* may be involved in signal transduction through its association with phenylpropanoid metabolism [54]. PPT is responsible for the supply of PEP to the shikimate pathway in the chloroplast stroma [55], [56]. The *cue1* mutant, which is defective in PPT and shows altered chloroplast development, has dark-green veins and light-green interveinal regions [57]. While this phenotype can be explained at least in part by a restriction in PEP supply to the shikimate pathway [57], the *cue1* mutant phenotype could not be rescued by the over-expression of a cauliflower PPT [58]. Grafting experiments involving *cue1* mutants suggested that leaf and root PPTs may fulfil different roles, with the leaf chloroplast PPT acting as a PEP importer while the root plastid PPT acts as an overflow valve [59].

While PPTs are present in both photosynthetic and heterotrophic tissues, another group of phosphate transporters, the glucose-6-phosphate/phosphate transporters (GPT), are found only in heterotrophic tissues [42]. The GPT group mediates the transport of glucose-6-phosphate, triose phosphates and phosphates into plastids [60]. The *Arabidopsis* genome contains two *GPT* genes, *AtGPT1* and *AtGPT2* [54]. *GPT1* is expressed in a similar manner to genes involved in starch metabolism [61]. The *Arabidopsis Atgpt1* mutants have severe defects in pollen development [62]. GPT2 plays a crucial role in the partitioning of glucose 6-phosphate between the plastids and cytosol during the transition from heterotrophic to autotrophic growth [63]. Over expression of *GPT2* led to an inhibition of carbon metabolism [64] and there was an early onset of senescence [65] although photosynthesis showed similar responses to increasing light intensities.

The xyulose-5-phosphate transporters (XPT) catalyse the exchange of xyulose-5-phosphate and to a lesser extent ribulose-5-phosphate for Pi [53]. These transporters link the pentose phosphate pathways in the plastids and cytosol, transporting metabolites for use in the Benson-Calvin cycle or other metabolic pathways [54], [66].

## 5 Transporters for the storage of carbohydrates in the vacuole

Just as triose phosphates are the main end-products of photosynthesis that are exported by chloroplasts, sucrose is the major export carbohydrate of leaves [67] and also an important signalling metabolite conveying information on resource availability throughout the plant [68], [69]. Sucrose synthesised in the cytosol is transported from the leaves to the phloem. Starch is synthesised and stored in chloroplasts during the light period and it is broken down to glucose and maltose at night and shipped to the cytosol for the synthesis of sucrose [70]. The dynamic synthesis and breakdown of starch, together with sucrose synthesis and export, ensures that the non-photosynthetic tissues receive a constant supply of carbohydrate to drive metabolism and growth in the light and in the dark. The key enzymes of the sucrose biosynthesis pathway, sucrose phosphate synthase (SPS) and sucrose phosphate phosphatase (SPP) appear to form a complex in the cytosol, allowing interactions between the proteins that not only influence the soluble carbohydrate pools but also modifies carbon partitioning to starch [71]. It is interesting to note that SPS has an N-terminal glycosyltransferase and a C-terminal phosphatase domain. This two domain structure bears a remarkable resemblance to that of the enzymes that catalyse the synthesis of another disaccharide, trehalose. Like sucrose, trehalose synthesis involves a two-step process, catalysed by trehalose-6-phosphate synthase (TPS) and trehalose 6-phosphate phosphatase (TPP). Trehalose-6-phosphate (T6P) is formed by TPS and is then dephosphorylated to trehalose by TPP, a reaction sequence that is relevant to the ways in which plants sense sucrose, as discussed later.

In addition to carbohydrate storage in leaves as starch, sucrose, hexoses and raffinose can also be stored in the vacuoles [71], which may aid the stabilisation of the organelles during adverse conditions, as well as into the guard cells in a light-dependent manner to influence stomatal aperture [72]. Sugars stored in the vacuole during the day are generally released and exported during the night [73]. Like excessive starch accumulation in chloroplasts, high leaf sucrose concentrations can inhibit photosynthesis [74]. In the case of sucrose, inhibition of photosynthesis is linked to sugar-mediated repression of the expression of photosynthetic genes [75].

Although the sucrose stored in leaf vacuoles represents only are small amount of the sucrose transported to the phloem, vacuolar sucrose levels can exert a strong effect on photosynthesis and stress tolerance. Moreover, vacuolar sucrose storage is particularly important in supporting the heterotrophic growth of reproductive tissues and developing embryos. The transport of sucrose within leaf mesophyll cells is driven by the concentration gradient between in the cytosol and vacuole [76]. Members of sucrose transporter (SUT) family transport sucrose across the tonoplast membranes. The tonoplast sucrose transporters in barley (HvSUT2) and *Arabidopsis* (AtSUT4) are able to transport sucrose when expressed in yeast [77], [78]. However, HvSUT2 may function to support the starch accumulation required for the embryo during grain development [77].

The *Arabidopsis* tonoplast monosaccharide transporter (TMT) family consists of three members, which are expressed in a tissue- and cell-specific manner. TMT1 is expressed in pollen, flowers and young developing tissues and TMT2 is expressed in young roots, floral tissues and mature leaves. In contrast, TMT3 transcripts are low in abundance, and are found only in seedlings, mature leaves and stamens. The expression of TMT1 and TMT2 is increased in response to drought and cold treatments, suggesting a role for these transporters in the response to environmental stresses [79]. A homologue of TMT was also found in the tonoplast fraction of barley mesophyll cells [74], suggesting that TMT may also have a role in sugar transport even under optimal growth conditions. More recently, the sugar beet transporter *BvTST2.1* was found to contribute to vacuolar sucrose uptake. This transporter operates as a proton antiporter, and has a high similarity to *Arabidopsis* TMT family amino acid sequences. This prompted the name change of the TMT family of transporters, to the TST family (tonoplast sucrose transporters) [80]. Glucose transporter 1 (*AtVGT1*) is localised on the tonoplast membrane in *Arabidopsis*. The *Atvgt1* mutants showed delayed flowering, together with a reduced capacity for viable seed production [81]. These data demonstrate that the sugars stored in the vacuole and their transport are essential for plant metabolism and related source/sink interactions.

## 6 Phloem loading

The distribution of resources between plant organs relies on the plant vasculature system comprising of xylem and phloem. The xylem is responsible for the transport of water and minerals collected from roots up to the shoots. The phloem is responsible for the transport of nitrogen- and carbon-containing compounds from source tissues such as leaves, to sink tissues. The phloem tissue consists of two cell types, sieve element (SE), responsible for nutrient conduction, and companion cell (CC), which provides metabolic support for the SE [82]. Together with the xylem, the phloem is an essential vascular network, providing carbon and nutrients to areas of the plants when needed.

The phloem is an important trafficking path in source/sink interactions linking key processes required for sugar signalling that not only controls the flow of sugars to developing organs, but also influences gene expression and hormone signalling throughout the plant [83]. Very few sugars that are synthesised in plants are transported long-distance through the phloem. Sucrose is the preferred transport form of carbon in most species. Thus, sucrose is the most abundant sugar found in the phloem in most plants [84]. The majority of sucrose produced in mesophyll cells is released into the apoplast and loaded into the sieve element (SE)/companion cell (CC) complexes of the phloem [82]. The SS/CC complexes are stacked longitudinally to form sieve tubes, which are able to transport sugars throughout the plant. Furthermore, there are at least three different but overlapping phloem types. These are the collection phloem, release phloem and transport phloem, the latter comprising the majority of phloem tissue. The collection phloem is located in smaller veins of the source leaves and is responsible for sucrose export. Release phloem is found in sink tissues (such as fruit, seeds and tubers) and is responsible for sucrose unloading. The transport phloem moves sucrose (and other solutes) around the plant, relying on osmotic pressures between source and sink to aid direction [83]. To date, three mechanisms for phloem loading have been described. In most species, phloem loading is apoplastic and facilitated by the membrane transporters described above, and transport is driven by the proton motive force [84], [85], [86], [87]. However, symplastic transport predominates, requiring high densities of plasmodesmata to facilitate sucrose movement to the phloem. Loading is thereafter driven by the sucrose concentration gradient between the mesophyll and phloem. This type of symplastic loading requires the maintenance of high sucrose concentrations in the mesophyll to maintain the downhill sucrose gradient [88], [89]. However, in some species, symplastic phloem loading in leaves is not directly apparent, despite high concentrations of sucrose in the mesophyll, no evidence of sucrose accumulation against a concentration gradient was found [90]. More recently a revised model of phloem loading has been described in rice whereby sucrose diffuses passively from the mesophyll through the plasmodesmata to the minor veins. Mesophyll sucrose concentrations are higher than the minor veins maintained by a tonoplast SUT transporter, which therefore acts a valve able to regulate sucrose flux into the phloem [88]. In some symplastic loaders, sucrose diffuses into the CCs of the minor veins from the mesophyll, where it is converted to stachyose and raffinose. These oligosaccharides are unable to diffuse back into the mesophyll and accumulate in the phloem, as a result of polymer-trapping [91].

Sucrose transporters play an integral role in apoplastic phloem loading and unloading, as well as in the exchange of sucrose between the plant and beneficial and/or parasitic symbiotic organisms [92]. Crucially, sucrose transporters play a key role in signal transduction between source and sink tissues [93]. Sucrose transporters belong to the major facilitator family, comprising 9 sucrose transporter genes (*SUCs* or *SUTs*) in *Arabidopsis*. Most SUTS are sucrose/H + symporters. However, other sucrose transporters are classified as sucrose facilitators (SUFs) because they catalyse a pH-independent and energy-dependent bi-directional transport of sucrose [94]. Phylogenetic analysis has been used to classify sucrose transporters into five clades: SUT1 (dicot-specific), SUT2 (found in monocots and dicots), SUT3 (monocot-specific), SUT4 (monocots and dicots) and SUT5 (monocot-specific) [84]. SUT1 clade and SUT3 clade members are typically expressed in the SE or CC or in both cell types. The SUT2 and SUT4 clades, which have a low affinity for sucrose, are generally expressed only in the plasma membrane of SEs. However, some SUT4 members have also been observed in the chloroplast and vacuole [84]. While the SUT5 clade, which was recently separated from the SUT3 clade remains poorly characterised, the gene encoding the rice SUT5 protein was reported to be highly expressed in sink leaves [88], [95].

In apoplastic loading/unloading species, sucrose is transported from the apoplast to the SE/CC by the SUT1/SUT3 (dicot/monocot) H +/sucrose co-transporter [96]. Null mutants of maize *sut1* had stunted growth, delayed flowering, and early senescence linked to a lower capacity for sucrose export [97]. Antisense tobacco, tomato and potato with low SUT1 showed similar phenotypes [98], [99], [100]. However, while decreased SUT1 expression in rice resulted in lower grain-filling capacity [101], [102], loss of the transporter did not lead to leaf carbohydrate accumulation or stunted phenotype [103]. In general, loss of *SUT1* function results in reduced sucrose transport while increased *SUT1* expression results in increased sucrose transport [84]. Taken together, these studies demonstrate the central role of *SUT1* in apoplastic loading in autotrophic/source tissues, and unloading in heterotrophic/sink tissues. While SUT1 is a key transporter in phloem loading and unloading, the other SUT proteins are important in cell-type specific sucrose transport particularly during reproductive development. For example, members of the SUT2 family are expressed in the phloem SEs and also in pollen. Loss of SUT2 function in tomato decreased pollen viability, through reduced sucrose uptake [99]. The rice SUT3 is also highly expressed in developing pollen [104]. SUT4 members are localised on the plasma membrane and the tonoplast membranes [105]. The rice *SUT4* is expressed during seed germination, as well as in pollen and in anthers at the post heading stage. *Arabidopsis SUT5*, which is expressed specifically in the endosperm, is considered to be a nutrient carrier of the filial tissues during early seed development [106]. It is important to note that although most sucrose transporters only have a high affinity for sucrose, some sucrose transporters, such as AtSUC2, can also transport other glycosides [107]. Although sucrose is the preferred plant transport form of carbon source, it is likely that sucrose derivatives are transported for use in other metabolic processes, or perhaps are used as signal molecules.

While the SUT super-family are major transporters involved in the translocation of sucrose between the apoplast and phloem, other transporters responsible for the movement of sucrose also fulfil important roles. For example, the SWEET transporters have been implicated in the transport of sucrose from the phloem parenchyma to the apoplast [108]. There are 17 SWEET family members in *Arabidopsis* that fall into four clades. In rice, there are 21 SWEET family members falling into the same four clades. The function of clade II and clade IV members remains to be identified, however members of clade I have been shown to mediate glucose import/export [93] and members of clade III preferentially transport sucrose across the plasma membrane [108]. There is growing evidence that SWEETs are bidirectional, pH-independent, low-affinity sucrose transporters, which operate through a uniporter mechanism of transport [109].

**Fig. 2:**
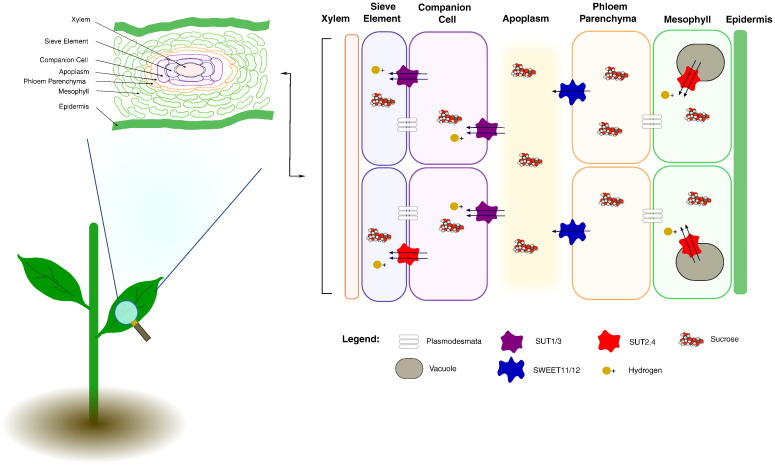
Apoplastic loading of sucrose from the mesophyll to the phloem in leaf tissue. Sucrose produced during photosynthesis is stored in the vacuole and transported into the mesophyll by SUT2/4. Sucrose moves to the phloem parenchyma through plasmodesmata where it is transported to the apoplast by SWEETS. SUT1/3 transport sucrose from the apoplast to the companion cell of the phloem and from the companion cell to the sieve element by plasmodesmata, SUT2/4 and SUT1/3. Arrows represent direction of transport.

The SWEET transporters are localised in the phloem parenchyma. GFP-tagged SWEET11 from *Arabidopsis* (AtSWEET11) was localised to the plasma membrane [108]. However, localisation in the phloem parenchyma remains to be confirmed. AtSWEET11 and AtSWEET12 share 88% identity at the amino acid level. There appears to be some functional redundancy in the SWEET protein family as the *Atsweet11* and *Atsweet12* mutants had no marked phenotype. However, *Atsweet11:12* double mutants had slower growth and showed carbohydrate accumulation in the leaves [108]. The phenotypes of the *Atsweet11:12* mutants were not as severe the *sut1* mutants, indicating that other SWEET proteins may compensate for the loss of SWEET11 and SWEET12 activity [109]. Studies on SWEETs and SUT1 have shown that apoplastic phloem loading occurs in two steps [107], [109]. Firstly, sucrose is exported from the phloem parenchyma to the apoplast by SWEETs. Secondly, sucrose is transported into the cells of the SE/CC complex from the apoplast by SUT1 (Fig. 2).

## 7 Starch accumulation

Excess carbohydrates are often stored as starch in source and sink tissues. Sucrose transported from leaves is used for the synthesis of starch at sites where carbohydrate storage is required, for example in seeds [110]. Starch accumulation in chloroplasts predominates during the day. While starch synthesis and degradation can occur simultaneously, starch degradation and remobilisation occur largely at night [110]. The *Arabidopsis* starch excess (*sex*) mutant was originally thought to have reduced expression of a chloroplast hexose transporter [111]. However, it was later found to have reduced expression of the starch granule R1 protein, which controls the phosphate content of starch [112]. Increased starch accumulation can be affected by maltose metabolism and transport. Maltose is accumulated in the chloroplasts during starch breakdown [113]. Maltose excess (*mex*) mutants, which are defective in the maltose transporter *MEX1,* were unable to convert starch into sucrose during the night, leading to increased starch accumulation in leaves [114]. Similarly, apple *mex1* mutants were unable to degrade starch during the night [115]. *MEX1* is expressed in mature apple leaves and also in sink tissues. The function of the *MEX1* transporter in sink tissues is unknown but it is likely to be required to support seedling growth, ensuring rapid use of stored carbohydrates to drive growth [115].

In addition to transporters, other proteins have been implicated in starch accumulation. *Tie-Dyed1* (*Tdy1*), which has only been described in grasses, is a transmembrane protein that promotes sucrose loading into the phloem [116]. Maize *tdy1* mutants are stunted with chlorotic leaves because of excessive leaf starch accumulation [86], possibly at the site of leaf veins [117], and produce oil droplets in the companion cells [118]. However, these mutants are not defective in phloem unloading, suggesting that *Tdy1* functions in carbon partitioning through the promotion of phloem loading [116]. Interestingly, the maize *sucrose export defective 1* (*sxd1*) mutant displays a similar phenotype to *tdy1* [117]. In addition, it was found that TDY1 and TDY2, a proposed callose synthase, may interact to promote symplastic transport in the phloem [118]. Since these transporters are specific to grasses, some phloem loading processes may be unique to monocots [116]. Further evidence of unique phloem loading process in monocots was described earlier in rice [88].

There may be differences in the types of sugar transporters used to support grain development in different species. For example, HvSUT1 has been implicated in seed starch accumulation. The expression of *HvSUT1* was directly associated with sucrose accumulation in the caryopses, where sucrose influx correlated with increased starch accumulation [77]. Further evidence for a role of SUT1 in grain filling has been obtained in rice [119], wheat [120] and barley [77]. These SUT1 transporters maintain the sugar supply to the developing grain by transporting sugar from the apoplast to the phloem [120]. In contrast, AtSUT5 appears to function in endosperm-specific sucrose transport in *Arabidopsis* [106]. The SWEET transporters are also important in grain filling in *Arabidopsis* [109]. The *sweet11;12;15* triple mutants showed severe defects in seed development producing lower weight wrinkled seeds, with reduced starch and lipid [109].

## 8 Trehalose metabolism and sugar signalling

**Fig. 3:**
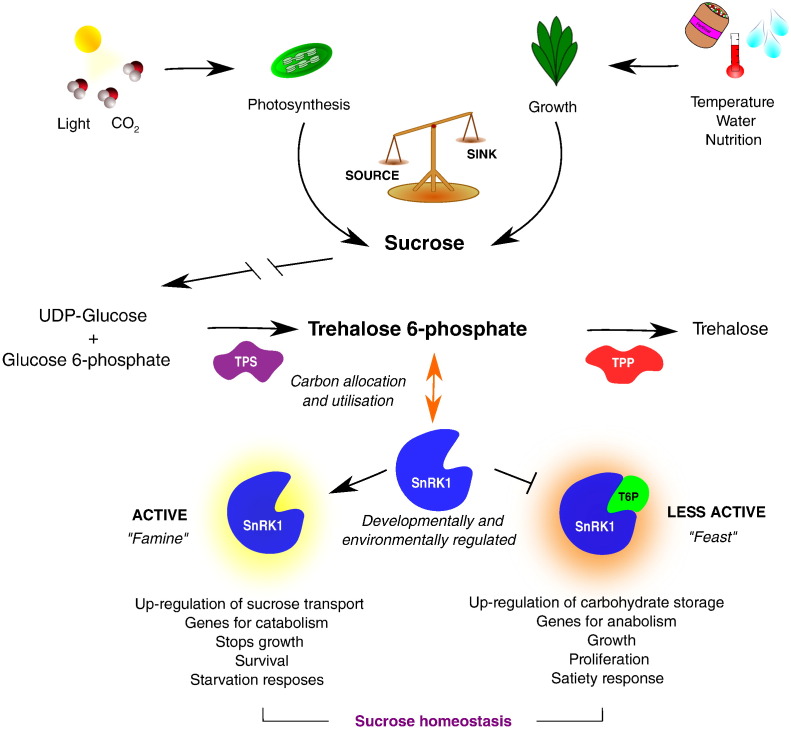
Sugar signalling via trehalose 6-phosphate (T6P)/SnRK1 interactions. The sucrose status of plant tissue is relayed through T6P to SnRK1. The activity of SnRK1 which is inhibited by T6P determines gene expression for starvation or satiety responses and maintains sucrose homeostasis. There is strong cell and developmental specificity of expression of genes regulating T6P content. Cell specific changes in T6P may enable modification of plant process and particularly productivity and resilience of crops.

Processes that consume sucrose are sensitive to stress-induced inhibition. For example, sucrose accumulation is observed in plants exposed to low temperatures [121], drought and salt stress [122], [123] and nutrient deficiency [124]. Sucrose is sensed by the plant directly, through the generation of hexoses and through sugar signals such as T6P (trehalose-6-phosphate) which relay the sugar status of the plant into mechanisms that enable adaptation to different environmental conditions (Fig. 2). Hexoses are produced only where sucrose is being metabolised, whereas T6P can be produced as a sugar signal wherever sucrose and the trehalose pathway are present. T6P is produced as an intermediate compound in the trehalose biosynthesis pathway. Briefly, UDP-glucose and glucose 6-phosphate are used to produce T6P catalysed by trehalose phosphate synthase (TPS), T6P is then converted into trehalose by trehalose phosphate phosphatase (TPP; Fig. 3). The flux of carbon into trehalose is four orders of magnitude less than into sucrose. Therefore, T6P synthesis will not cause depletion of UDPG and G6P pools. Whether the levels of UDPG and G6P are important regulators of T6P synthesis is unknown but there is correlation between the abundance of these metabolites and that of T6P. However, T6P is synthesised in actively growing tissues where sucrose is metabolised to yield these substrates [124]. Accumulating evidence shows that T6P levels are most related closely to sucrose pool size [124], [125].

**Fig. 4:**
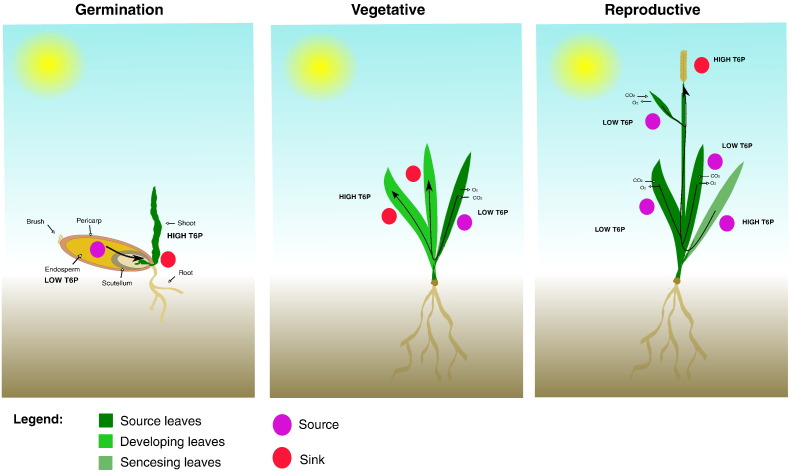
Source-sick and T6P-related processes throughout the life cycle. Changes in T6P accumulation, and source-sink relationships affect cellular processes in seed germination, vegetative and reproductive plant growth. Source tissues generally have a low T6P content, and sink tissues a high T6P content. Arrows indicate direction of sucrose transport.

T6P is a universal signal of sucrose concentration in plants [121], [124], [126], [127], [128]. The abundance of T6P does not respond to the levels of sugars such as glucose [121]. Thus, T6P is considered to be a specific signal for sucrose availability. T6P levels are controlled largely by the activity of *TPS1*. *Arabidopsis* mutants lacking the trehalose phosphate synthase gene, *tps1* are embryo lethal [129]. T6P inhibits Sucrose non-Fermenting Related Kinase 1 (SnRK1). Inhibition of this major signalling component results in metabolic reprogramming at sucrose levels above 3 μmol g^− 1^ FW in *Arabidopsis* seedlings [128] i.e. about 3 mM sucrose on a whole tissue basis. This sucrose levels results in the accumulation of T6P to about 1 μM on a whole tissue basis. Hence, physiologically-relevant T6P concentrations are between 1 and 10 μM. SnRK1 is inhibited over this concentration range, for example 50% inhibition is produced at 5.4 μM T6P [128]. Relatively small changes in T6P concentrations within this range may therefore give rise to large changes in SnRK1 activity [130] resulting in the metabolic reprogramming of hundreds of genes involved in growth and defence [131], [132]. In the absence of T6P, SnRK1 regulates the expression of a different subset of genes that are involved in catabolism rather than anabolic processes. This regulation is important for stopping growth when sucrose is in short supply, thus preventing starvation and death. Therefore, the combination of T6P and SnRK1 activity is integral in overall plant growth and development (Fig. 4). Glucose 1-phosphate (G1P) and glucose 6-phosphate (G6P) also inhibit SnRK1, but less potently than T6P (at levels of 480 μM, and > 1 mM respectively). However, when these inhibitors are combined with T6P, a synergistic effect is observed with G1P and an additive with G6P [128]. These effects provide considerable flexibility in the regulation of SnRK1. Strong inhibition of SnRK1 may only occur under sucrose-replete conditions, as proposed by Lunn et al. [125]. However, many environment stresses cause sucrose accumulation. Correlations between T6P, sucrose levels and the expression of SnRK1 marker genes have been observed under different growth conditions, such as cold, nitrogen deficiency and following sucrose feeding [128]. T6P promotes growth only when other conditions are not limiting. Hence, T6P functions as part of an integrated network involving sucrose and hormones that regulate growth [133]. The activation of gene expression in response to sucrose accumulation following exposure to cold stress prepares the plants for rapid growth “in anticipation” of return of warmth [128]. This may be an important adaptive response to unseasonal cold.

Large differences in synonymous and non-synonymous TPS and TPP substitutions have been observed in *Arabidopsis* [134]. These genes have strong cell-specific expression profiles and are induced differentially by environment and also by sugars [133], [134], [135]. The regulated expression of TPSs and TPPs is part of the plant response to fluctuations in sucrose supply. The regulation expression of different gene family members is driven by the requirement to optimise T6P in different cell types for certain environments.

## 9 Applications of sugar signalling in agriculture

**Fig. 5:**
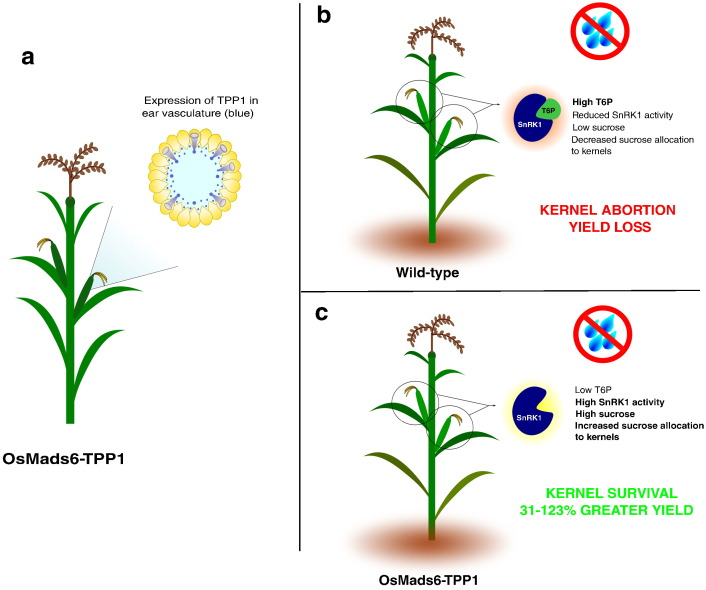
OsMADS6-TPP maize. Expression of TPP in female reproductive tissues of maize leads to increased yield in drought-stressed conditions. TPP is expressed in maize ear vasculature (a). In wild type plants, drought stress causes yield loss (b), in OsMADS6-TPP maize, yield loss due to drought-stress is decreased, resulting in higher yield in comparison to wild-type during drought-stress (c).

Manipulations of T6P levels have provided a new paradigm for crop improvement [122]. For example, a rice TPP linked to a MADS6 promoter was recently expressed in maize. MADS6 is expressed during the flowering period and plays an essential role in endosperm nutrient accumulation in ear nodes, ear vasculature and spikelet tissues [136], [137]. Drought at flowering can have a large effect on crop yields. Hence, manipulations have been sought that maintain the flow of sucrose to developing female reproductive tissues during drought [138], [139], [140]. MADS6-TPP expression decreased T6P levels in female reproductive tissues allowing increased sucrose availability in spikelets leading to improved harvest index. Yields were increased significantly (by up to 123%) by MADS6-TPP expression in rigorous field trials over a number of years, which involved multiple sites with a range of environments including soils with water deficits. The largest yield increases were observed under severe drought, however yield improvements were also seen under non-stressed conditions [122] (Fig. 5). T6P may regulate sugar levels as part of a homeostatic mechanism that ensures that sucrose does not accumulate to excessive levels or falls to very low levels, in a mechanism that is analogous to the control of blood glucose levels in animals by glucagon and insulin [125]. T6P was shown to perturb this homeostatic mechanism and to alter sucrose levels in the MADS6-TPP study [122].

Recent studies have confirmed the central role of T6P in managing whole plant carbon budgets and stress responses [141]. For example, a TPP gene was shown to be essential for anaerobic germination under flooding. In this case metabolism of T6P by TPP was perceived as a starvation signal, which enhanced starch mobilisation to drive the growth of the germinating embryo and elongating coleoptiles. Anaerobic germination tolerance enables uniform germination and seedling establishment under submergence. Hence, rice can be directly seeded rather than the current labour-intensive planting methods. It may be possible to harness the ability of T6P to function as both a starvation and satiety signal by increasing T6P. Decreasing T6P could be advantageous at other times. Driving down T6P levels may be effective in cells involved in sucrose transport as already demonstrated in maize and in germinating rice seeds [122], [141]. In other cell types, for example those that are actively converting sucrose to starch or oils, an increase in T6P levels may be effective in ensuring optimal gene expression for biosynthetic processes. Since large changes in yield can be achieved through one transgene which results in small changes in the target protein and in T6P levels, tailoring T6P metabolism could therefore make an important contribution to the improvement global food security.

SnRK1 may not be the only target of T6P. In mature leaves T6P does not inhibit SnRK1 because of different composition of SnRK1 complexes between heterotrophic and autotrophic tissues [128] and yet T6P regulates starch metabolism in rosette leaves through redox regulation of AGPase [140] and significantly through starch breakdown [141]. How T6P mediates these effects is not known. Similarly, T6P is necessary for leaf senescence [142]. Remarkably this may be due to the regulation imparted by T6P early in leaf development possibly through SnRK1. T6P is also part of the network that regulates flowering, providing information to plants concerning sucrose availability for flower development [143].

## 10 Concluding remarks

Plant metabolite transporters are crucial to inter-pathway regulation, cell to cell communication, and source-sink interactions. The sophisticated vasculature of plants is rich in transporters, which regulate the partitioning of resources and signals between source and sink organs. Carbon metabolism and transport is at the heart of whole plant communication and underpins key agricultural traits such as flowering, productivity and stress tolerance [122], [144]. Sugar signalling even exerts a key influence over processes such as apical dominance [145], which was once thought to be the exclusive domain of phytohormone regulation. The cell autonomous expression of trehalose pathway genes allows the fine tuning of specific responses to sucrose availability. T6P not only fulfils a major role in signalling cellular carbon status, but it is also a determinant of sink strength. It is therefore an attractive target for increasing yield and yield resilience. T6P exerts its effects through SnRK1 [96], which like sucrose is a conserved central regulator of plant metabolism. Similarly to sucrose, trehalose can be transported throughout the vascular system [135], however relatively little is known about plant trehalose and T6P transporters. At an intracellular level T6P is mainly localised in the cytosol, however is also present in the chloroplasts [124]. Again, little is known about T6P transport between cellular compartments.

An important consideration when aiming to improve crop yields is whether the yield itself is source or sink limited, which appears to be species-dependent. In potatoes for example, source capacity limits yield by up to 80% under glasshouse conditions [146]. In contrast, the yield of wheat seeds is largely dominated by sink activity [147]. Yield improvements can only come from a much deeper understanding of the equilibrium between source and sink tissues that intrinsically involves assimilate transport considerations at a whole plant level [148]. Future increases in crop yields are likely to come from improvements in transporter functions in sources and sinks. Manipulation of sucrose transporters, for example SUT1 and SWEETs, may have a dramatic effect on sucrose remobilisation and source/sink relationships underpinning plant growth and development. This may result in greater understanding of the source-sink relationship, and in-turn facilitate the development of sustainable, high-yielding crops.

## Conflict of interest

There is no conflict of interest.

## Transparency document

Transparency document.
